# Development and Validation of a Magnetic Resonance Imaging-Based Machine Learning Model for TMJ Pathologies

**DOI:** 10.1155/2021/6656773

**Published:** 2021-07-05

**Authors:** Kaan Orhan, Lukas Driesen, Sohaib Shujaat, Reinhilde Jacobs, Xiangfei Chai

**Affiliations:** ^1^OMFS IMPATH Research Group, Department of Imaging & Pathology, Faculty of Medicine, University of Leuven and Oral & Maxillofacial Surgery, University Hospitals Leuven, Leuven, Belgium; ^2^Department of DentoMaxillofacial Radiology, Faculty of Dentistry, Ankara University, Ankara, Turkey; ^3^Ankara University Medical Design Application and Research Center (MEDITAM), Ankara, Turkey; ^4^Huiying Medical Technology Co., Ltd., Room C103, B2, Dongsheng Science and Technology Park, HaiDian District, Beijing City 100192, China

## Abstract

The purpose of this study was to propose a machine learning model and assess its ability to classify TMJ pathologies on magnetic resonance (MR) images. This retrospective cohort study included 214 TMJs from 107 patients with TMJ signs and symptoms. A radiomics platform was used to extract (Huiying Medical Technology Co., Ltd., China) imaging features of TMJ pathologies, condylar bone changes, and disc displacements. Thereafter, different machine learning (ML) algorithms and logistic regression were implemented on radiomic features for feature selection, classification, and prediction. The following radiomic features included first-order statistics, shape, texture, gray-level cooccurrence matrix (GLCM), gray-level run length matrix (GLRLM), and gray-level size zone matrix (GLSZM). Six classifiers, including logistic regression (LR), random forest (RF), decision tree (DT), *k*-nearest neighbors (KNN), XGBoost, and support vector machine (SVM) were used for model building which could predict the TMJ pathologies. The performance of models was evaluated by sensitivity, specificity, and ROC curve. KNN and RF classifiers were found to be the most optimal machine learning model for the prediction of TMJ pathologies. The AUC, sensitivity, and specificity for the training set were 0.89 and 1, while those for the testing set were 0.77 and 0.74, respectively, for condylar changes and disc displacement, respectively. For TMJ condylar bone changes Large-Area High-Gray-Level Emphasis, Gray-Level Nonuniformity, Long-Run Emphasis Long-Run High-Gray-Level Emphasis, Flatness, and Volume features, while for TMJ disc displacements Average Intensity, Sum Average, Spherical Disproportion, and Entropy features, were selected. This study has proposed a machine learning model by KNN and RF analysis on TMJ MR images, which can be used to classify condylar changes and TMJ disc displacements.

## 1. Introduction

Temporomandibular joint (TMJ) problems are general in the general population and can affect up to one-third of all adults at some stage in their life. In previous population-based studies, temporomandibular disorders (TMD) as well as subgroups of these are often evaluated through clinical examination [1–3]. Magnetic resonance imaging (MRI) is a common diagnostic method of choice for TMD which serves a gold standard for examining the disc status, especially for soft tissue pathologies [4].

MRI examination of TMJ is usually performed using conventional sequences such as T1-weighted (T1W) and proton-weighted or T2-weighted (T2W) pulse sequences. It should be stated that TMJ is considerably smaller compared to other joints, thereby, utilization of conventional diagnostic methods make it difficult to detect joint inflammatory changes before the morphological changes [5].

The evaluation of an MR image is generally subjective, and the interpretation can be changed due to interpreter experience level, MR sequences, etc. The observers can also make different diagnoses for the same patient depending on the examination conditions and imaging modality. Thus, it is essential to establish a standardized MRI outcome for appropriate diagnosis for repeatability and reproducibility of diagnosis [6]. In addition to this, MRI interpretations are still falling short of showing a clear association with reported symptoms [7]. Furthermore, the impact of potential imaging findings on treatment choice and clinical outcome is still controversial, suggesting that the depiction of TMJ in clinical routine is still unsatisfactory and may benefit from further optimization [8]. Moreover, it was also indicated that, often, there is still an unclear correlation between clinical signs and symptoms and imaging findings in all TMD patient groups [9, 10].

Machine learning (ML) is a subfield of AI in which rather than explicit programming of instructions, the machine learns how to accomplish a task by mathematical analysis of datasets provided [11–13]. Medical image computing benefits from advances in machine learning to develop data-driven model-based image analysis strategies that are less biased by heuristic assumptions about the appearance of the objects in the images [14]. Studies about mathematical models based on AI techniques to support certain diagnoses can be found in the literature [15–17].

Applications of machine learning to dental imaging have been relatively sparse. Only limited studies were done for radiomic features, especially for TMJ. A recent investigation has shown that CBCT Texture Analysis can be used for differentiating mandibular condyle changes [18]. However, to the best of our knowledge, no study was done on TMJ MRI images. Thus, the purpose of this study was to propose a machine learning model and assess its ability to classify TMJ pathologies on magnetic resonance (MR) images.

## 2. Material and Methods

The Research Ethics Committee UZ/KU Leuven (Reference no.: MP010867) approved this retrospective analysis of anonymous data and waived the requirement for informed consent.

### 2.1. Patients and Data Management

A total of 214 TMJs from 107 patients (34 male and 73 female; mean age: 38 years ± 17.97; range: 19-74 years) were included in this study. All patients were examined clinically for TMJ disorders according to “Diagnostic Criteria for Temporomandibular Disorders for Clinical and Research Applications” [19] by the same clinician. After each examination, the patients underwent bilateral TMJ MRI. Inclusion criteria involved patients with anterior disc displacement with and without reduction (Table 1). The exclusion criteria for MRIs which were not diagnostically suitable for evaluation include motion artifacts, patients with sideway-partial or posterior disc displacements, and syndromic disease or history of trauma.

The MRI of TMJs was taken bilaterally for all participants. The images were taken with 1.5 T imaging units (Signa Horizon, GE Electric, Milwaukee; Gyroscan Intera, Philips Medical Systems, Washington; Magnetom SP4000, Siemens, Erlangen) with the help of dual-surface coils (3-inch and 6 × 8 cm surface coils). All patients underwent imaging in the axial, sagittal, and coronal planes using fast spin-echo sequences (FSE). The images were taken in the closed, partially opened, and maximally opened mouth positions to detect disc displacements.

T1-, T2-, and proton density-weighted images were taken from all patients with similar TR and TE values in different MR machines; for Signa Horizon (GE Electric, Milwaukee) and for Magnetom SP4000 (Siemens, Erlangen, Germany) T1-weighted images were taken with TR = 150, TE = 4.2 while bilateral sagittal and coronal proton density-weighted images and T2-weighted images were taken with TR = 2500, TE = 17 and TR = 2500, TE = 102, respectively, with 10 cm field of view, 192 × 256 matrix, NEX = 2, bandwidth = 15.6 kHz, and 3 mm slice thickness. For Gyroscan Intera (Philips Medical Systems, Washington), T1-weighted images were taken with TR = 300, TE = 16 while bilateral sagittal and coronal proton density-weighted images and T2-weighted images were taken with TR = 2000, TE = 19 and TR = 2000, TE = 80, respectively, with the 10 cm field of view, 256 × 128 matrix, NEX = 2 bandwidth = 15.6 kHz, and 3 mm slice thickness.

The disc displacement of the TMJs was classified as normal, anterior disc displacement with reduction, and anterior disc displacement without reduction again according to Orhan et al.'s study [6]. Two radiologists separately evaluated and interpreted the images twice without knowledge of the prevailing clinical conditions of the patients. Moreover, TMJs were classified as normal (without any osseous change) and with osseous changes (flattening, erosive changes in the morphology of the articular surfaces, anterior osteophytes, and/or subchondral lacunas) and were classified as degenerative joint diseases (ART) [3]. When the assessments differed, a consensus was reached through a repeated evaluation and a discussion between the radiologists. The conditions for consensus were 3 anterior disc displacement without reduction cases and 2 erosive changes which were verified in this session.

### 2.2. Data Management

Radiomics is an emerging field that converts imaging data into a high dimensional mineable feature space using a large number of automatically extracted data-characterization algorithms. Thus, a radiomics platform (Huiying Medical Technology Co., Ltd., China, http://en.huiyihuiying.com/) was used to manage imaging data, clinical data, and subsequent radiomics statistics analysis. These radiomics platforms have the potential to uncover the distinctive imaging algorithms to quantify the state of diseases, and thereby provide valuable information for personalized medicine. Moreover, they can measure features in an imaging exam that include intensity, shape, texture, wavelet, and LOG features to build predictive or prognostic noninvasive biomarkers or imaging modalities [13]. This platform can be used for the extraction of radiomics features from 2D and 3D images and binary masks on different imaging modalities such as CT and MRI.

### 2.3. Imaging Segmentation

TMJ condyles and discs were delineated on the MR images manually by a resident and senior radiologist independently (LD and KO) who were blinded to the clinical information of the patients. The software allows drawing contours using a Lasso tool that can draw out a manually shaped area defined by the mouse. When the tool travels, it can select objects within the contours delineated and allows adjusting the borders. All contours were then reviewed again and evaluated together for final adjustments in consensus.

**Figure 1:**
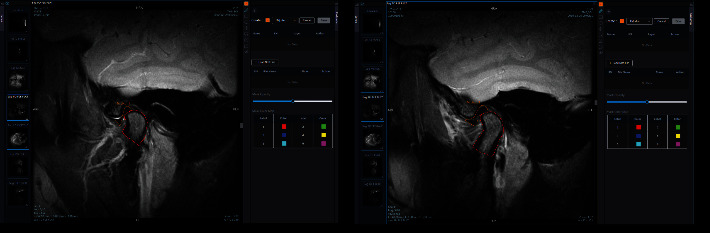
MRI images showing the tracings of TMJ disc and condyle in a radiomic platform.

In each TMJ, 4 VOIs (volume of interests) were delineated from MR images, Thus, for each patient, 4 right and 4 left VOIs were defined from MR scans. In total, 856 condyles and disc VOIs were manually traced from 107 patients' scans (214 TMJs) (Figure 1).

### 2.4. Feature Extraction and Selection

In total, 90 radiomic features were identified from VOIs of MR images using the radiomics platform. These radiomic features were under the first-order, shape, and texture classifications. In particular, the texture features containing gray-level cooccurrence matrix (GLCM), gray-level run length matrix (GLRLM), and gray-level size zone matrix (GLSZM) were used. Meanwhile, intensity and texture features were calculated on the original image and derived images, obtained by applying several filters such exponential, logarithm, square, square root, and wavelet (wavelet-LHL, wavelet-L, wavelet-HLL, wavelet-LLH, wavelet-HLH, wavelet- HHH, wavelet-HHL, and wavelet-LLL). Features comply with definitions as defined by the Imaging Biomarker Standardization Initiative (IBSI) [20]. To reduce the dimensionality of features, variance threshold methods were used to gradually select the optimal features. A variance threshold was also applied (variance threshold = 0.8) to reduce the features.

### 2.5. Consensus Clustering

A consensus clustering was also used to cluster the radiomic features extracted from the training sets TMJ condyle and disc. Consensus clustering is a resampling-based clustering methodology, which quantifies the consensus between several clustering iterations and provides means to estimate the number of clusters that best fit the data [21]. It is also a method of finding clusters that are more stable and less sensitive to starting values based on a membership principle. It considers multiple input clusterings where items have been clustered repeatedly in order to remove bias [22].

The first task to build consensus clustering involves the construction of an *n* × *n* “agreement matrix” based on input clustering results. Thus, an estimation was made for the range for the appropriate number of clusters using *k*-means clustering.

*k*-means clustering is one of the most commonly used unsupervised machine learning algorithm for partitioning a given data set into a set of *k* groups, where *k* represents the number of groups prespecified by the observers. It classifies objects within the same cluster as similar as possible (i.e., high intraclass similarity), whereas it classifies objects from different clusters as dissimilar as possible (i.e., low interclass similarity). In *k*-means clustering, each cluster is represented by its center which corresponds to the mean of points assigned to the cluster [23].

After building up the range, the number of clusters that gave the highest median cluster consensus over all clusters was chosen. Cluster consensus was defined as the average consensus between all pairs of features belonging to the same cluster.

Cluster consensus (range [1,–1]) indicates the robustness (stability) of a cluster over resampling. A qualitative categorization of cluster stability was defined as follows: consensus < 0.5, poor stability; 0.5 ≤ consensuses < 0.75, moderate stability; and consensus ≥ 0.75, high stability. Consensus clustering was performed using the Radcloud platform.

### 2.6. Statistical Analysis

All statistical analyses were performed on the Radcloud platform. Computer-generated random numbers were used to assign 80% of the VOIs to the training data set and 20% of VOIs to the validation data set. Six classifiers, including logistic regression (LR), random forest (RF), decision tree (DT), *k*-nearest neighbors (KNN), XGBoost, and support vector machine (SVM) were used for the model building which could predict the TMJ pathologies. The performance of models was evaluated by sensitivity, specificity, and ROC curve. The optimal cutoff value was selected as the point when the sensitivity plus specificity was maximal. The AUC and prediction accuracy were calculated in both the training and validation sets. Then, we used four indicators as well including *P* (precision = true positives/(true positives + false positives)), *R* (recall = true positives/(true positives + false negatives)), F1-score (F1 − score = *P*∗*R*∗2/(*P* + *R*)), and support (total number in test set) to evaluate the performance of classifiers.

## 3. Results

### 3.1. Subjective Evaluation

The distribution of disc displacements in the patient group (*n* = 107) according to the examination criteria is shown in Table 2. MRI examination showed that 78.5% of the patients had disc displacements. It also showed that 46 joints (46/214) were normal in the sagittal slices although they were symptomatic clinically. Disc displacements were found in 168/214 (ADDwR+ADDwoR) in the whole group. ADDwR was the most common with 74.4% (125/168), while ADDwoR was in 25.6% of the patients (43/168). Normal function with the normal osseous condition was noted in all the joints that had discs in the superior position. Moreover, subjective evaluation of TMJ condyle showed that ART was more common in patients with ADDwoR than other types of disc displacement. ART was observed in 49 TMJs with ADDwR (39.2%) and 27 TMJs with ADD woR (62.7%).

### 3.2. Machine Learning and Radiomic Results

**Figure 2:**
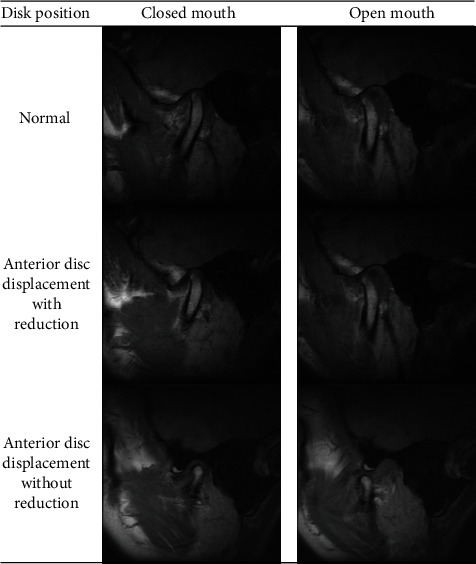
MRI images demonstrating normal and anterior disc locations with and without reduction.

**Figure 3:**
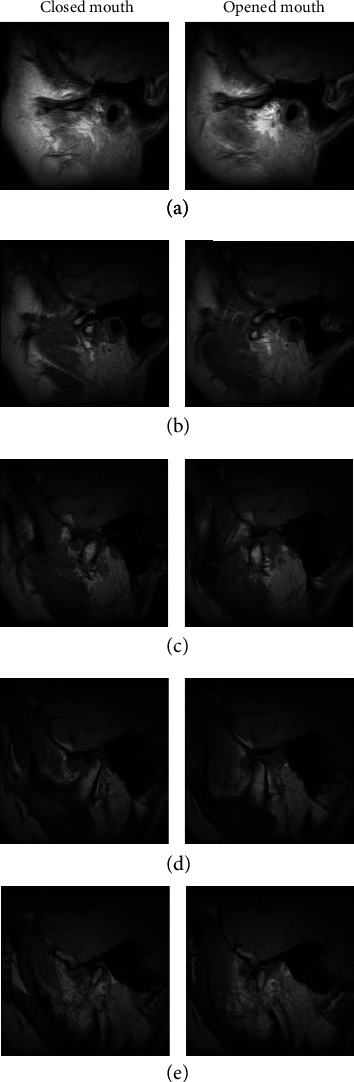
MRI images demonstrating degenerative joint disease in series.

For further analysis with machine learning, disc displacements were classified for machine learning as follows: normal and anterior disc displacement (ADDw+ADDwoR). Condyles were classified as follows: (Figure 2) normal (without any osseous change) and ART (with any osseous changes) (Figure 3).

Of all 90 identified radiomic features, the Radcloud platform selected 56 features that can be calculated and associated with features for TMJ pathologies (both for condyle and TMJ disc changes) from MR images (Table 3).

**Figure 4:**
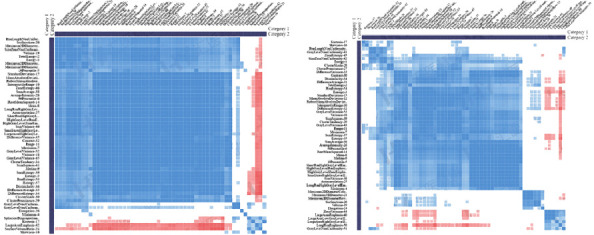
Cluster consensus maps of (a) TMJ condyle and (b) TMJ disc. Note that condyle category 1 means normal (without any osseous change) while category 2 means ART (with any osseous changes); TMJ disc category 1 means normal, while category 2 means anterior disc displacement.

Figure 4 depicts a CI heat map of radiomic features in the validation cohorts of TMJ condyle and TMJ, and it was observed that 56 features had significant prognostic performance (CI > 0.5) to differentiate from normal to ART and from normal to ADD.

These features were grouped into three groups. Group 1 (first-order statistics) consisted of 18 descriptors that quantitatively delineated the distribution of voxel intensities within the MR image through commonly used and basic metrics. Group 2 (shape- and size-based features) contained 8 two- and three-dimensional features that reflect the shape and size of the region. Calculated from gray-level run length and gray-level cooccurrence texture matrices, there were 30 textural features that can quantify region heterogeneity differences.

**Figure 5:**
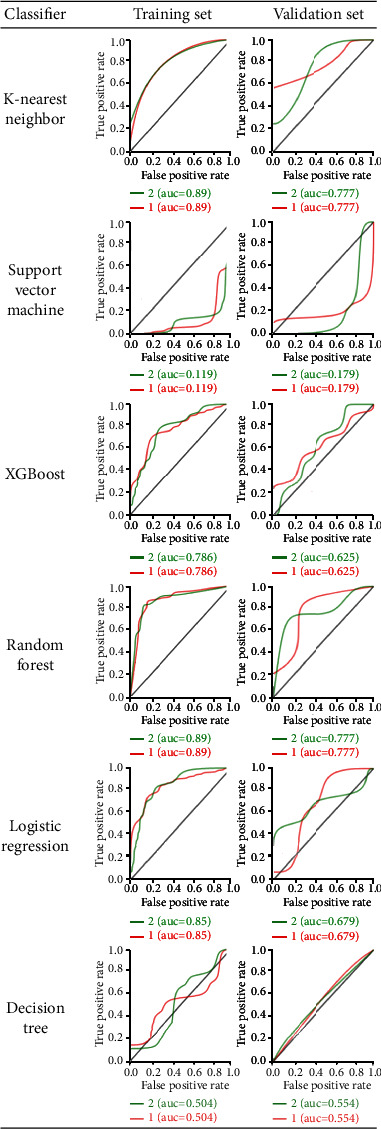
ROC analysis by six classifiers of the training set and the validation set for mandibular condyle: “1” indicates normal; “2” indicates degenerative joint diseases.

ROC curve analysis results are shown in Figure 5 for the training set and validation set. For the selection of radiomic features, the AUC of KNN and RF machine learning methods was high, with a range of 0.89 and 0.77 for the training set and validation set, respectively.  Table 4 summarizes the four indicators (precision, recall, F1-score, and support) for six classifiers. For condylar changes in KNN and RF, classifiers were the best methods in training and validation sets on diagnostic performance by four indicators. Moreover, several radiomic features were selected to identify condylar changes as Large-Area High-Gray-Level Emphasis, Gray-Level Nonuniformity, Long-Run Emphasis, and Long-Run High-Gray-Level Emphasis, and also Flatness and Volume for shape radiomics.

**Figure 6:**
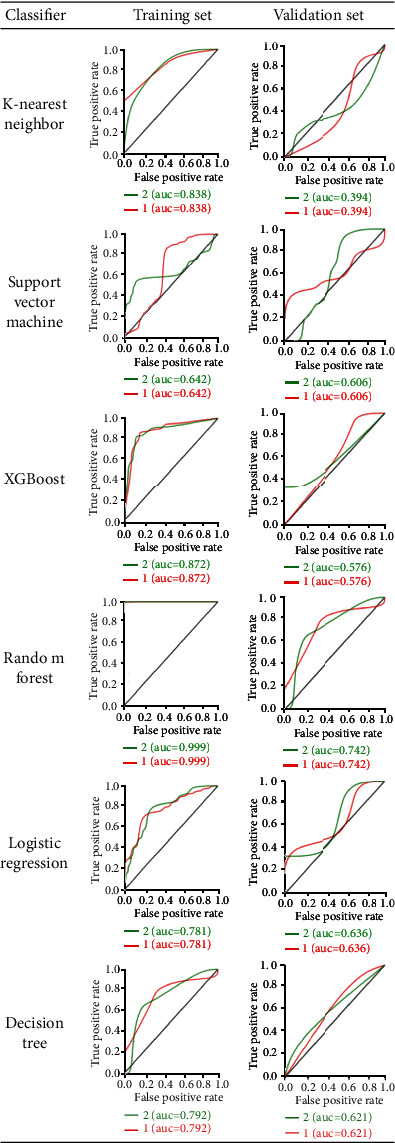
ROC analysis by six classifiers of the training set and the validation set for TMJ disc: “1” indicates normal. “2” indicates anterior disc displacement.

**Figure 7:**
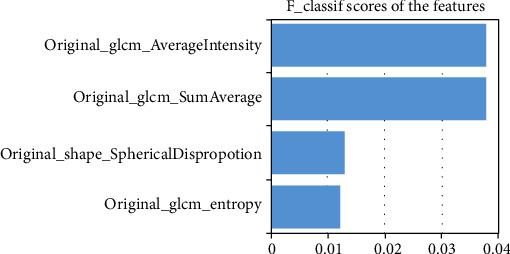
The selection of *K* best methods to further evaluate the radiomic with *F* classifier scores for TMJ disc displacements.

**Figure 8:**
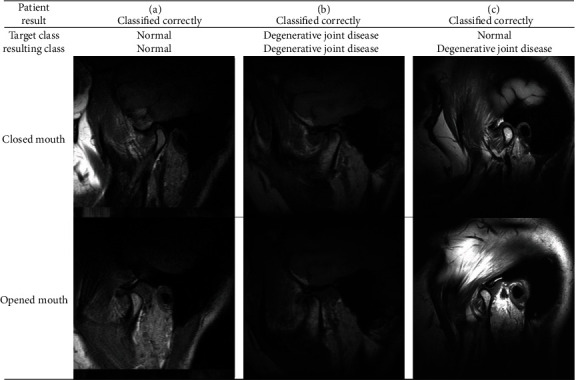
Examples of classifications made with a random forest model for degenerative joint disease.

**Figure 9:**
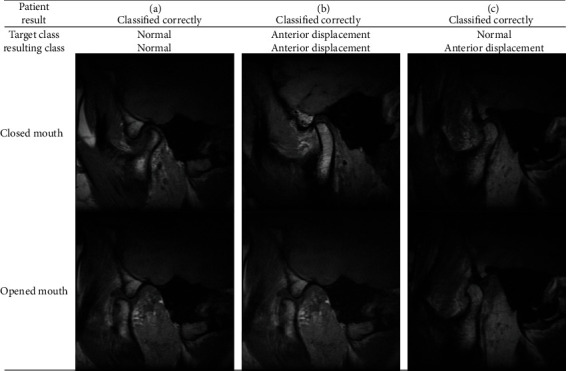
Examples of classifications made with the random forest model to determine the disc location.

For disc displacement, ROC curve analysis results are shown in Figure 6 for training and validation sets for disc displacement. The AUC of the RF machine learning method was medium to high, with a range of 0.99 and 0.74 for the training set and validation set, respectively. The variance threshold method was used to select the*K*best methods from 56 features. In the final stage, the features that did not reach statistical significance dropped, and finally, 4 features were selected, namely, Average Intensity, Sum Average, Spherical Disproportion, and Entropy (Figure 7). For disc displacements, the RF classifier was the best method in the validation set on diagnostic performance by four indicators (Table 5). KNN and RF were found to be the best methods for identifying the mandibular condyle changes (Figure 8), whereas the RF classifier was the best machine learning approach for quantifying TMJ disc placements (Figure 9) on MR images.

## 4. Discussion

The use of a machine learning system as an AI approach with the application of radiomic features is limited in the dentomaxillofacial field. Hence, in this study, a machine learning approach to identify condyle bone changes and disc displacements was presented.

Several previous studies indicated the use of gray-level texture analysis for TMD use using CT/CBCT images. Caramella et al. [24] demonstrated gray-level texture analysis should be taken carefully since there has been a difference in CT or CBCT acquisition protocols and reconstruction algorithms. A recent study by Bianchi et al. [18] standardized these variables and validated them using different software. They found that the variables that did not present such distinction between the sample averages were mainly correlation for GLCM and GLRLM, i.e., Long-Run Emphasis and Long-Run High-Gray-Level Emphasis. Based on their results, they concluded that CBCT Texture Analysis can be used for differentiating mandibular condyle changes. Recent studies also showed that GLCM and GLRLM textural features are potential diagnostic markers of TMJ osteoartrosis [25, 26]. The previous studies indicated a high and significant correlation for bone morphometry and all the GLCM features, with an exception for two GLRLM variables, namely, Gray-Level Nonuniformity and Short-Run Emphasis in Bianchi et al.'s study [18]. In this study, in line with previous studies, a high correlation was found for all the GLCM features and correlation was found for GLSZM features such as Large-Area High-Gray-Level Emphasis and Gray-Level Nonuniformity and GLRLM features Long-Run Emphasis and Long-Run High-Gray-Level Emphasis. Our results also showed the Gray-Level Nonuniformity feature for identifying the condylar changes in contrast to Bianchi et al.'s [18] study. However, it should be stated that previous studies were conducted utilizing CT/CBCT imaging; however, in this study MRI, was used for radiomic identification. For TMJ disc displacement, so far no study attempted to identify the MRI radiomic features. Based on the results of this study, Average Intensity, Sum Average, Spherical Disproportion, and Entropy can be used for disc displacement identification.

Different machine learning strategies, such as *k*-nearest neighbors, support vector machines, or random forest decision trees, can be applied to construct the mapping of a given training set and a given set of features. During training, the parameters that define the mapping, whose representation depends on the chosen learning strategy, are iteratively refined such that estimation performance is maximized on the training set itself; together with this evaluation, the difference between the given ground truth for each image and in the training set can be evaluated. The KNN classifier is one of the popular image classification algorithms that directly calculates image to image distances in comparison with other classifiers that need a training phase to calculate the distance between an image and a class [27]. Meanwhile, random forests or random decision forests are an ensemble learning method for classification, regression, and other tasks that operate by constructing a multitude of decision trees at training time and outputting the class that is the mode of the classes (classification) or mean prediction (regression) of the individual trees [28].

In a recent study [27], researchers classified TMJ condyles as normal and TMD and used CBCT images. They found that KNN has been the best classifier in detecting patients from healthy individuals with a 92% accuracy, 94% sensitivity, and 90% specificity. Our study is in line with Haghnegahdar et al.'s study [29].

There are several limitations to the current study. Although the results of this study are significant, the results of this study remain suggestive and have to be confirmed by a study with a larger sample size especially predicting various disc displacements such as partial or sideway disc displacements with and without reduction. In this study, we did not attempt to differentiate ADDw and ADDwoR cases, and further studies should be conducted for identifying the reduction state. On the other hand, other clinical parameters have to be correlated with radiomic features to understand whether these parameters are related to the TMJ pathologies such as biomarkers. This study may also be conducted using magnetic resonance imaging (MRI) radiomic features.

Considering that machine learning is a subdivision of artificial intelligence (AI), and taking into account that AI software in general is only able to “learn” by itself after being induced to that, possible diagnostic divergences made by humans may constitute a bias to the software learning process. More robust retrospective and prospective studies will be required to ensure clinical applicability. Future studies will be aimed at exploring different approaches in presenting radiologists with model output assessments, including TMD risk calculations. Moreover, validation studies need to be carried out for assessing the effect of different MRI protocols on feature detection and AI performance. Another opportunity would be to apply similar modeling techniques to routine diagnostic CBCT, aiding detection and management of TMD pathologies.

## 5. Conclusions

This study has proposed a machine learning model by KNN and RF analysis on TMJ MR images, which can classify the condylar changes and TMJ disc displacements. This study also demonstrated that the combination of specific MRI-based radiomic features with image variables can predict TMJ pathologies.

## Figures and Tables

**Table 1:** The classification of study group using DC/TMJ.

*Normal*: in the closed-mouth position, the posterior band of the disc is located superior to the condyle in which the posterior band of the TMJ disc is at the apex of the condylar head (12 o'clock position). When the jaw is opened, the disc remains interposed between the osseous components and moves anteriorly in a synchronized fashion. In the coronal plane of imaging, the disc is centered perfectly on the condylar head.

*Anterior disc displacement with reduction (ADDwR)*: an intracapsular biomechanical disorder involving the condyle-disc complex. In the closed-mouth position, the disc is in an anterior position relative to the condylar head, and the disc intermittently reduces with the opening of the mouth. When the disc does not reduce with the opening of the mouth, an intermittent limited mandibular opening occurs. When a limited opening occurs, a maneuver may be needed to unlock the TMJ. Medial and lateral displacement of the disc may also be present. Clicking, popping, or snapping noises may occur with disc reduction.

*Anterior disc displacement without reduction (ADDwoR)*: an intracapsular biomechanical disorder involving the condyle-disc complex. In the closed-mouth position, the disc is in an anterior position relative to the condylar head, and the disc does not reduce with the opening of the mouth. Medial and lateral displacement of the disc may also be present. This disorder is associated with a persistent limited mandibular opening that does not reduce with the clinician or patient performing a manipulative maneuver.

**Table 2:** The distribution of the disc positions.

Disc positions	Number of TMJs	%
Normal	46	21.5
^∗^ADDwR	125	58.4
^∗^ADDwoR	43	20.1
Total	214	100

^∗^ADDwR: anterior disc displacement with reduction. ^∗^ADDwoR: anterior disc displacement without reduction.

**Table 3:** Radiomic features selected for quantifying the heterogeneity differences.

Radiomic group	Associated filter	No. of features (*n* = 56)	Radiomic features

First-order statistics	None	18	Energy, total energy, entropy, minimum, 10 percentile, 90 percentile, maximum, mean, median, interquartile range, range, mean absolute deviation, robust mean absolute deviation, root mean square, standard deviation, skewness, kurtosis, variance

Shape	None	8	Volume, surface area, surface volume ratio, spherical disproportion, maximum 3D diameter, maximum 2D diameter column, maximum 2D diameter row, elongation

Texture features	GLCM	15	Autocorrelation, average intensity, cluster prominence, cluster shade, cluster tendency, contrast, difference average, difference entropy, difference variance, dissimilarity, entropy, sum average, sum entropy, sum variance, sum squares

Texture features	GLSZM	8	Large-area emphasis, gray-level nonuniformity, size zone nonuniformity, gray-level variance, zone entropy, high-gray-level zone emphasis, small-area high-gray-level emphasis, large-area high-gray-level emphasis

Texture features	GLRLM	7	Gray-level nonuniformity, run length nonuniformity, gray-level variance, run entropy, high-gray-level run emphasis, short-run high-gray-level emphasis, long-run high-level emphasis

Label: GLCM = gray-level cooccurrence matrix; GLSZM = gray-level size zone matrix; GLRLM = gray-level run length matrix.

**Table 4:** Evaluation for diagnostic performance by four indicators for mandibular condyle: precision, recall, F1-score, and support in the training set. “1” indicates normal. “2” indicates degenerative joint diseases.

		Indicators	KNN	SVM	XGBoost	RF	LR	DT
Training	1	Precision	0.83	0.79	0.91	0.95	0.81	0.88
		Recall	0.92	1.00	0.98	1.00	0.98	0.98
		F1-score	0.88	0.88	0.95	0.97	0.89	0.94
		Support	53	53	53	53	53	53
	2	Precision	0.50	0.00	0.90	1.00	0.67	0.80
		Recall	0.29	0.00	0.64	0.79	0.14	0.74
		F1-score	0.36	0.00	0.75	0.88	0.24	0.76
		Support	14	14	14	14	14	14

Testing	1	Precision	0.81	0.78	0.81	0.78	0.78	0.70
		Recall	0.93	1.00	0.93	1.00	1.00	0.86
		F1-score	0.87	0.88	0.87	0.88	0.88	0.83
		Support	14	14	14	14	14	14
	2	Precision	0.50	0.00	0.50	0.00	0.00	0.33
		Recall	0.25	0.00	0.25	0.00	0.00	0.25
		F1-score	0.33	0.00	0.33	0.00	0.00	0.29
		Support	4	4	4	4	4	4

LR: logistic regression; RF: random forest; DT: decision tree; KNN: *k*-nearest neighbors; XGBoost; SVM: support vector machine.

**Table 5:** Evaluation for diagnostic performance by four indicators set for TMJ disc: precision, recall, F1-score, and support in the training set. “1” indicates normal. “2” indicates anterior disc displacement.

		Indicators	KNN	SVM	XGBoost	RF	LR	DT
Training	1	Precision	0.84	0.77	0.88	0.999	0.80	0.78
		Recall	0.95	1.00	0.90	0.999	0.93	0.89
		F1-score	0.89	0.87	0.89	0.999	0.86	0.86
		Support	40	40	40	40	40	40
	2	Precision	0.71	0.00	0.64	1.00	0.50	0.54
		Recall	0.42	0.00	0.58	1.00	0.25	0.28
		F1-score	0.53	0.00	0.61	1.00	0.33	0.48
		Support	12	12	12	12	12	12

Testing	1	Precision	0.77	0.79	0.85	0.79	0.79	0.73
		Recall	0.91	1.00	1.00	1.00	1.00	0.81
		F1-score	0.83	0.88	0.92	0.88	0.88	0.77
		Support	11	11	11	11	11	11
	2	Precision	0.00	0.00	1.00	0.00	0.00	0.50
		Recall	0.00	0.00	0.33	0.00	0.00	0.33
		F1-score	0.00	0.00	0.50	0.00	0.00	0.40
		Support	3	3	3	3	3	3

## Data Availability

All data of the present article are available on request by contacting the corresponding author.
